# Antithyroid Antibodies and Thyroid Function in Pediatric Patients with Celiac Disease

**DOI:** 10.1155/2015/276575

**Published:** 2015-02-19

**Authors:** Derya Kalyoncu, Nafiye Urganci

**Affiliations:** ^1^Department of Pediatrics, Sisli Etfal Training and Research Hospital, 34270 Istanbul, Turkey; ^2^Division of Pediatric Gastroenterology, Sisli Etfal Training and Research Hospital, 34270 Istanbul, Turkey

## Abstract

*Objective*. Aim of the study was to determine the prevalence of autoimmune thyroid disease, persistence of antithyroid antibodies, effect of gluten-free diet, and long-term outcome of thyroid function in pediatric patients with celiac disease (CD). *Methods*. 67 patients with CD aged from 1 year to 16 years were screened for thyroid antithyroperoxidase, antithyroglobulin and anti-TSH receptor antibodies, serum free triiodothyronine, free thyroxine, and thyroid-stimulating hormone (TSH) at diagnosis and during follow-up. *Results*. None of the patients had antithyroid antibodies at diagnosis. Antithyroid antibodies became positive in 16.4% of the patients (11/67) 2 to 3 years after the diagnosis of CD. Clinical hypothyroidism was observed only in 3 of 11 CD patients with positive antithyroid antibodies (27.2%). The antithyroid antibodies positive and negative patients did not differ significantly according to compliance to GFD (*P* > 0.05). A statistically significant difference was observed only in age, in which the patients with positive antithyroid antibodies were younger than the patients with negative antithyroid antibodies (*P* = 0.004). None of the patients had any change in their thyroid function and antibody profile during their follow-up. *Conclusion*. Antithyroid antibodies were detected in younger pediatric patients with CD and the prevalence of antithyroid antibodies did not correlate with the duration of gluten intake.

## 1. Introduction 

Celiac disease (CD) is an immune-mediated disease triggered by an environmental agent, gluten, in genetically predisposed individuals, characterized by villous atrophy of the proximal small intestine and malabsorption. Gluten induces the production of specific autoantibodies directed against tissue transglutaminase not only in the small intestine but also in other tissues.

The association between CD and other autoimmune diseases such as type 1 diabetes mellitus, autoimmune thyroiditis, and other endocrine diseases has been reported in many studies in both children and adults [1–19]. It has been suggested that prolonged exposure to gluten in CD may promote the development of other autoimmune diseases by causing production of autoantibodies [2]. This coexistence is thought to be partly due to HLA-DQ2 and DQ8 haplotypes which are overrepresented in many autoimmune diseases [20, 21] and the inheritance of these haplotypes [19]. Besides similar HLA haplotypes, association with the gene encoding cytotoxic T-lymphocyte-associated antigen-4 (CTLA-4), a candidate gene for conferring susceptibility to thyroid autoimmunity, also has been reported [19, 22–24]. Among autoimmune disorders, thyroid abnormalities were reported to be 26.2% [13], 37.6% [12], and 41.1% [14] in pediatric patients with CD.

This study was carried out to determine the prevalence of autoimmune thyroid disease, the relationship between antithyroid antibodies and gluten-free diet (GFD), persistence of antithyroid antibodies during follow-up, and long-term outcome of thyroid function in children with CD.

## 2. Materials and Methods

Sixty-seven children with CD diagnosed and followed up between 1999 and 2011 at Division of Pediatric Gastroenterology of Sisli Etfal Training and Research Hospital (Istanbul, Turkey) were evaluated prospectively. The diagnosis of CD was based on ESPGHAN criteria [25]. The histopathological changes of small intestinal biopsies were graded according to a modified Marsh classification [26]. Informed consents were obtained from all of the parents before the procedures. During follow-up, dietary compliance to GFD was evaluated on every visit by the same physician and celiac disease activity was monitored by measurement of antibodies against transglutaminase and endomysium. A family history of autoimmune diseases was determined among first-degree relatives of all patients.

Baseline thyroid antithyroperoxidase (TPOAb), antithyroglobulin (TGAb) and anti-TSH receptor (TRAb) antibodies, serum free triiodothyronine (FT3), free thyroxine (FT4), and thyroid-stimulating hormone (TSH) were examined in all patients. FT3, FT4, and TSH were detected by enhanced chemiluminescence immunoassay (ECLIA) using commercial kits (normal values, FT4: 0.86–1.78 ng/dL; FT3: 3.2–6.8 pmol/L; TSH: 0.7–5.97 mU/L). Serum antithyroid antibody titers were detected using chemiluminescent immunoassay (CLIA) and radioimmunoassay (RIA). Antithyroid antibodies and thyroid function tests were measured regularly once every year during follow-up. In children with increased antibody titers, thyroid ultrasonography was performed.

The diagnosis of autoimmune thyroid disease was based on positivity of antithyroid antibodies and diffuse or irregular hypoechogenicity of the thyroid gland on ultrasound examination. Euthyroidism was defined as normal TSH and FT4 levels. Clinical hypothyroidism was defined as an increase in TSH serum concentration and a significant decrease in FT4 level. Subclinical hypothyroidism was recognised by TSH elevation with normal concentrations of circulating thyroid hormones (FT4 and FT3). Hyperthyroidism was defined as suppressed, usually undetectable serum TSH concentration and increased FT4 and FT3. Subclinical hyperthyroidism was noted by decreased TSH serum levels and normal FT4.

### 2.1. Statistical Analysis

Statistical analyses were performed using SPSS 11.0 software (SPSS Inc., Chicago, IL, USA). Results were expressed as means ± SD for quantitative variables and proportions for categorical variables. The analysis was conducted using Fisher's exact test, chi-square test, and ANOVA to analyze qualitative variables. *P* values of <0.05 were considered statistically significant.

## 3. Results

The age of patients ranged from 1 year to 16 years (mean 6.77 ± 4.64), and male : female ratio was 0.59. No patient had a family history of autoimmune diseases. 16.4% of the patients (11/67) had antithyroid antibodies which became positive 2 to 3 years after the diagnosis of CD. There was no serologic or clinical sign of another autoimmune disease in patients with CD except for two patients with type 1 diabetes mellitus. These two patients had negative antithyroid antibodies. In two patients, autoimmune disease (type 1 diabetes mellitus) was diagnosed before CD while all of the autoimmune thyroid disease diagnoses were subsequent to the diagnosis of CD. The baseline demographic and clinical characteristics of the cases are shown in Table 1.

When compared with antithyroid antibodies negative patients, the patients with positive antithyroid antibodies were younger at diagnosis and the difference was statistically significant (*P* = 0.004). The antithyroid antibodies positive and negative patients did not differ significantly in gender, weight, height, clinical presentation, and histological type according to the modified Marsh criteria and compliance to GFD (*P* > 0.05) (Table 1).

Clinical hypothyroidism was observed in 3 of those 11 CD patients with positive antithyroid antibodies (27.2%) but none of the patients with negative antithyroid antibodies. Hyperthyroidism was diagnosed in none of the patients. All of the patients except 3 with hypothyroidism had normal thyroid function (euthyroidism) at diagnosis and none had any variation in their thyroid function and antibody profile during their follow-up. All of 3 patients with hypothyroidism were compliant with GFD. Ultrasonography showed abnormal thyroid pattern characterized by diffuse hypoechogenicity in 3 patients with hypothyroidism and normoechoic sonographic pattern in other patients.

Of 11 patients with persistently positive antithyroid antibody titers, 8 (72.7%) remained consistently euthyroid during the follow-up and subclinical hypothyroidism was detected in none of them. Three patients with clinical hypothyroidism became euthyroid with levothyroxine therapy given.

## 4. Discussion

The association between CD and other autoimmune disorders such as type 1 diabetes mellitus, autoimmune thyroid disease, and other endocrine diseases is well established in many studies [1–19]. Early identification of autoimmune disorders in patients with CD is important since it may be useful in the control of autoimmune disease itself, as well as in the prevention of long-term complications of CD.

An increased prevalence of antithyroid antibodies has been reported in patients with CD [12–14]. 16.4% of our patients had antithyroid antibodies in this study, similarly as reported in previous studies [8, 11, 16, 27] and lower than that obtained by Forchielli et al. [12], Ansaldi et al. [13], and Kowalska et al. [14].

Although some authors disagree [28, 29], it has been reported that the prevalence of autoimmune disorders in CD increased with increasing age at diagnosis [1, 15], which means late diagnosis of CD causes longer exposure to gluten and higher incidence of autoimmune diseases. Oderda et al. [16] reported that untreated children with antithyroid antibodies at diagnosis were significantly older, suggesting that the duration of gluten exposure may be another important risk factor for the development of autoimmunity. In contrast with these studies, CD patients with positive antithyroid antibodies were significantly younger than the patients with negative antithyroid antibodies in our study (mean age, 3.22 ± 2.21 versus 7.47 ± 4.69 years, resp., *P* = 0.004).

Cosnes et al. [30] demonstrated that CD patients who were diagnosed earlier in life and had family history of autoimmunity were most at risk for autoimmune disorders. In particular, the first-degree relatives of CD patients have an increased risk of autoimmune diseases, most likely connected with unrecognized subclinical or silent forms of CD [31, 32]. A family history of autoimmune diseases was determined in none of our patients. It has been suggested that GFD was not sufficient to suppress thyroid autoimmunity when it has already started and early diagnosis of CD and an early gluten withdrawal may be preventive for thyroiditis [16]. CD patients with antithyroid antibodies were diagnosed in earlier ages than the patients with negative antithyroid antibodies in our study. Antithyroid antibodies became positive in 11 patients 2 to 3 years after the diagnosis of CD while 63.6% of them were compliant with GFD.

At initial phase, signs and symptoms of thyroid disease are usually absent and TSH may be normal and anti-TPO antibodies may be positive [31]. Because there is possibility of worsening of thyroid function over time, early recognition of thyroid dysfunction is necessary to prevent the negative effects of hypothyroidism on growth, metabolic function, and celiac disease. As the disease progresses, the immune response to the target cell destroys the endocrine gland, leading to hypofunction. Thus, subclinical hypothyroidism (TSH elevation with a normal FT4) and then clinical hypothyroidism (increase in TSH serum concentration and decrease in FT4 level) appear [31]. It has been suggested that long-term follow-up of euthyroid patients with positive autoimmune thyroid serology would be advisable to establish whether antithyroid antibodies could demonstrate a higher propensity for thyroid involvement [13]. The antithyroid antibodies were positive in 12.5% (8/64) of our euthyroid patients with CD and no change in their thyroid function was observed during their follow-up.

Although some of the previous studies have reported that duration of gluten exposure in CD does not correlate with the risk for autoimmune disease and that gluten withdrawal did not protect from autoimmune disease [8, 13, 29], several reports suggested that adherence to GFD was associated with a decreased risk of subsequent autoimmune disease and antithyroid antibodies tend to disappear during GFD [1, 11, 30]. Guariso et al. [17] have concluded that GFD seems to produce a favourable effect on the previously present clinical autoimmune disease and to prevent the development of new clinical autoimmune disease but does not affect the onset of potential autoimmunity. Our results showed that there was no significant correlation between compliance to GFD and autoimmune thyroid disease and thyroid dysfunction. 63.6% (7/11) of the CD patients with positive antithyroid antibodies and all of the patients with hypothyroidism (3/3) were compliant with GFD; thus gluten withdrawal did not protect them. Antithyroid antibodies persisted despite GFD during follow-up. 44.7% (30/67) of our patients were noncompliant with GFD and only 4 of them developed antithyroid antibodies.

The correlation between the degree of histological changes and the appearance of autoantibodies could not be compared in this study, because, according to the modified Marsh criteria, the disease was severe (IIIb/IIIc) in all of the patients enrolled in this study.

In conclusion, we only observed significant difference between presence of antithyroid antibodies and younger age in patients with CD. The prevalence of antithyroid antibodies did not correlate with the duration of gluten intake and compliance to GFD in this study. Thyroid function should be assessed in all children with CD at diagnosis and during follow-up. Further, larger, prospective studies with longer follow-up are needed to clarify the clinical significance of antithyroid antibodies in pediatric patients with CD and the effect of GFD and other factors on the development of autoimmune thyroid disease.

## Figures and Tables

**Table 1:** Characteristics of pediatric patients with celiac disease.

CD patients	Thyroid antibodies (+) (*n* = 11)	Thyroid antibodies (−) (*n* = 56)	*P*
Age at diagnosis (year)	3.22 ± 2.21	7.47 ± 4.69	0.004
Gender (male/female)	0.57 : 1 (4/7)	0.6 : 1 (21/35)	1.00
Weight			
<3rd percentile	6 (54.5%)	29 (51.7%)	1.00
>3rd percentile	5 (45.4%)	27 (48.2%)	0.82
Height			
<3rd percentile	6 (54.5%)	27 (48.2%)	0.75
>3rd percentile	5 (45.4%)	29 (51.7%)	0.70
Clinical presentation			
Typical	9 (81.8%)	32 (57.1%)	0.18
Atypical	2 (18.1%)	24 (42.8%)	0.12
Compliance to GFD			
Compliant	7 (63.6%)	30 (53.5%)	0.74
Noncompliant	4 (36.3%)	26 (46.4%)	0.54
Duration of follow-up	8.05 ± 3.6	6.7 ± 4.1	0.31
Laboratory findings			
TSH (mU/L)	2.92 ± 1.56	2.99 ± 1.73	0.95
FT4 (ng/dL)	1.10 ± 0.31	1.37 ± 1.39	0.58
FT3 (pmol/L)	3.28 ± 0.98	3.25 ± 0.45	0.61

GFD: gluten-free diet; TSH: thyrotropin (thyroid-stimulating hormone); FT4: free thyroxine; FT3: free triiodothyronine.

*P* < 0.05 is statistically significant.
